# A systematic review of food-environment interactions in catchment models

**DOI:** 10.1007/s44279-025-00355-9

**Published:** 2025-09-16

**Authors:** Luke Roche, Cathal O’Donoghue, David Styles

**Affiliations:** University of Galway, Galway, Ireland

**Keywords:** Catchment models, Food-environment interactions, Land use, Ecosystem services, Agricultural production, Water quality, Environmental management

## Abstract

This paper provides a systematic review of food-environment interactions in catchment models, focusing on the complex relationships between land use, ecosystem services, and agricultural production. The review highlights the importance of catchment-scale models in understanding the impact of land use on local ecosystems, particularly in relation to water quality, biodiversity, and soil services. By examining various international catchment-scale models, with an emphasis on the micro and macro disparities, the paper identifies key methodological lessons and future opportunities to enhance these frameworks for more effective policy design and environmental management. The review is structured around a conceptual framework that categorises models into environmental models, which focus on ecosystem dynamics, and physical models, which examine structural and material aspects of land use systems, as well as human dimensions, with a specific focus on reducing net emissions and improving land productivity. This relationship is described through a conceptual framework. The paper also emphasises the significance of spatial and temporal factors in these models, noting gaps in the literature limited integration of food production into catchment models, underrepresentation of localised/catchment-level modelling, data limitations, particularly lack of georeferenced micro-level data, inadequate incorporation of climate change scenarios and temporal variability, weak integration of biophysical, economic, and social factors, insufficient analysis of policy and governance impacts at catchment scale and lack of farm-specific, actionable recommendations. The research highlights the critical need for enhanced data accessibility, environmental model maintainability, standardised land use variable definitions, and improved georeferencing in land use modelling. The findings emphasise the importance of long-term projections, integration of social, economic, and biophysical factors, and open data initiatives to bolster essential research infrastructure and foster stakeholder engagement for more effective agricultural policy and environmental management.

## Introduction

Land use ecosystem services play a critical role at local and catchment scales by supporting ecological balance and resource availability. Food production in pastoral landscapes amplifies these impacts, influencing water quality, biodiversity, landscape value, and soil health, all of which is dependent on how agricultural practices are managed. However, the delivery of these complex combinations of goods and ecosystem services is challenging given the wide variety of influences; bio-physical, policy and behavioural. Land use models at catchment scale can play a key role in understanding these factors and can assist in designing policy and incentive structures that can improve the delivery of these food-environment interaction outcomes at a catchment level. Given the growth of the land use challenges as they relate to environmental and social issues, the literature on catchment scale land use modelling and analysis has grown in recent years. This review focuses on food–environment interactions, which focuses on the relationships between agricultural production systems, ecological processes, and catchment-scale dynamics. While the broader food environment (as defined in food systems literature) refers to consumer-facing spaces such as markets, supermarkets, or informal food outlets [22], this paper emphasises the ecological and production-side interactions that link food systems with land use, ecosystem services, and resource management.

The aim of this review is to classify the existing land use models in the field of agricultural economics and food environment interactions at a catchment scale and to address key topics such as: what methodologies are most effective for integrating food-environment interactions in catchment-scale models? How do we identify methodological gaps and limitations in existing models, particularly concerning their ability to represent food production systems alongside other ecosystem services. The paper aims to provide insights and recommendations for advancing the development of integrated, localised models that can better inform policy and decision-making in the context of sustainable agriculture and land-use management. While global land-use models effectively address broad public goods issues like climate change, they lack the localised focus needed to analyse complex, site-specific interactions between biophysical, social, and economic factors [49]. Existing catchment-scale research often overlooks the role of food production systems, focusing instead on water-related dynamics and neglecting broader ecosystem services and human factors. (Addressing this gap is critical for developing more holistic models that inform sustainable agricultural practices and land-use policies. Limited data availability, the complexity of integrating multi-dimensional factors, and the absence of comprehensive food-environment interaction models further highlight the need for research in this area.)

Food-environment interactions take place at a localised/catchment scale, where modelling enables assessmentof social, economic, and biophysical aspects of natural resource management [51], all strongly linked to definitions of food environment interactions [22]. Land use models at a catchment scale are a subset of wider literature on land use models. This is supported throughout research and emphasised that data limitations often play a key role in the specification of any model [11]. They are part of a spectrum of models that look at human-biophysical interactions in relation to land that span large scale global integrated assessment land use models to more granular single catchment models understanding for example local hydrological-human activity interactions. While global land use models are useful for global public goods related issues like climate change which allow the development of strategies, where multidimensional considerations are essential. Adapting to climate change, catchment scale models are more appropriate for local public good analysis and should be encouraged in literature [27].

One of the common themes throughout the review papers reference the complexities of land use models. It is believed that ‘complexity arises from both human decision making and the explicitly spatial aspects of the landscape environment’ [58]. They place an emphasis on the interconnected relationships that exist among and between agents, and their biophysical environment over time and space. However, the increase in computing power has empowered more detailed analysis and allowed for additional complexity [73]. The growth in complexity has also allowed for scaling differences between policy and intrinsic scales to be as small as possible, which has helped in avoiding model induced errors. The complexity is conceptualised in the field, where they use a three-layered dimension to categorise and summarise models; space, time and human choice [1]. Space considers the physical layout and features of the land. Time looks at how land use changes over different periods and human choice recognises that people’s decisions play a significant role in shaping land use. By focusing on spatial resolution in microsimulation modelling through a systematic review, the index system employed explores the relationship between modelling methods and food-environment applications.

It is believed that modelling land use dynamics should consider six key factors [74]: (1) Multi-scale dynamics, involving nesting of scales and cross-scale interactions. (2) Economic, social, ecological, environmental, historical, and cultural factors. (3) Spatial interaction and neighbourhood effects, resulting from spatial autocorrelation, environmental gradients, and land use distribution. (4) System changes, which are often nonlinear with critical thresholds. (5) Past changes influencing the present and future. (6) Understanding interactions among subsystems in the system studied. By recognising these key terms, this may allow for a classification between different approaches to land use modelling. The inclusion of such key factors is essential to model for the realistic for the construction and evaluation of present and future land use scenarios. This paper looks at the underrepresented area of research in land use modelling of integrated land use models at catchment scale. Catchment level research can have a significant impact on the performance of land zone but a lack of research results in land use change impacts being poorly understood at catchment scale [64]. This paper aims to contribute by investigating this gap in the literature.

Land-use change is a widespread phenomenon that researchers increasingly study through modelling methods, particularly statistical approaches, to analyse and compare trends at both micro and macro scales [56]. These models play a vital role in understanding the causes and consequences of land-use dynamics, thereby supporting policymakers in making informed decisions. Previous studies highlight the diversity of land-use change modelling methods and emphasise that selecting an appropriate model scale is a complex challenge, requiring consideration of the model’s objectives and the specific research problem being addressed [51, 64, 75]. Building on this, the present review engages more directly with catchment-scale models, with a specific focus on food–environment interactions.

The number of meta-analyses focusing on integrated agricultural land use modelling has been steadily increasing in recent years, as is reflected in several internationally published papers [75, 50, 15]. These papers all share the common theme of linking the localised land use to the wider context goals and policies associated with the climate and sustainability-oriented objectives and the associated decision-making methods and tools on an international level. However, a significant number of the review articles published in this field can be attributed to catchment scale modelling which is orientated around the localised water systems [64, 32, 18, 3]. This review highlights the need for more of a focus on land-use modelling at the intersection of agriculture and economics. Although many of the same issues relate to the wider context of sustainability and meeting climate action targets, there remains a specific gap in modelling approaches that integrate agricultural production within catchment-level analyses. Existing land-use models often emphasise biophysical processes and face significant data limitations as localised models require detailed, in depth information that is often not openly available [1].

As a result land-use models remain limited in both number and scope. Specifically, few models adequately represent food production systems, most focus on water quality while overlooking other ecosystem services and human dimensions [26, 60]. Addressing these gaps plays a big role in addressing these omitted areas. This review aims to make that contribution.

## Conceptual framework

**Fig. 1:**
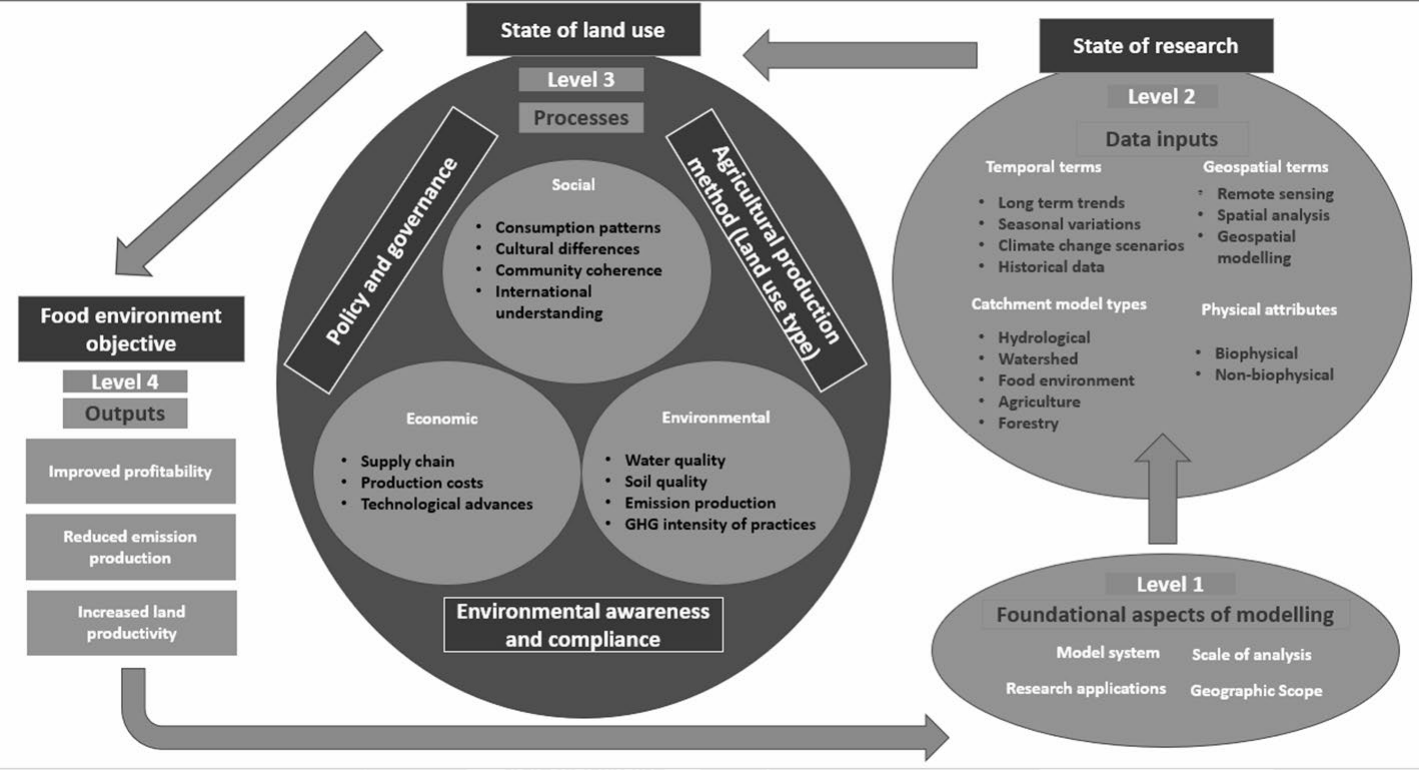
Food Environment Interactions Conceptual Framework four-level analytical structure used to classify land-use models in relation to food–environment interactions at catchment scale

Land use for food production is both a key component of land-use systems and a central part of food systems at the interface between actors that provide inputs in food systems and those that process and consume food [53]. Land use plays a key role in a number of societal structures, including food security, economic development and ecological stability [39]. All of which are deeply intertwined with land use behaviour. Land use changes have very important implications for future changes in Earth’s climate [1]. When conducting analysis using land use models, the scale of analysis plays a central role in the process. It is stated that model structure clearly represents the hierarchical organisation of land-use systems, outlining specific roles of each type of analysis regarding the output [74]. As localised land use models gather data specific to an area, this allows for greater detail to be included at a household level, with more flexibility and attention being paid to location specific variables such as land quality, policy influence, and societal variables. At the regional level, the effects of land-use change on natural resources can be determined by a combination of land use change analysis and specific models to assess the impact on natural resources [74]. The methods and data section will outline the scientific procedure and initial assumptions, while the results and discussion section will present key findings. This structured approach ensures transparency and reproducibility in the research process. Figure 1 presents the conceptual framework that guided this review. It outlines the four levels of analysis (the heuristics) used to classify and examine the models identified in the literature. The framework illustrates how methodological choices (state of research) and food-use dynamics (state of land use) are organised across four levels: foundational assumptions, data inputs, processes, and outputs, which cycle back into the state of research again.

A conceptual framework in the context of a review of food-environment interaction modelling highlights the key variables in the models, while linking them to an overarching research aim. This structure shows the analytical steps of the review rather than results, ensuring readers can follow how models were examined in relation to food–environment interactions.In this review, the framework is organised into four levels as follows: the foundational aspects of modelling (level 1), data inputs (level 2), processes (level 3) and outputs (level 4). The arrows indicate general linkages between levels of modelling, rather than strict causal feedback loops The framework outlined. does not represent results but rather the structure of analysis applied to this study. Reducing net emissions is a key objective in driving shifts in agricultural land use methodologies, with the impact largely dependent on farming activities, crop and livestock productivity, food consumption patterns, and agricultural trade dynamics [76], this conceptual framework depicts four main levels that connect academic research in land use modelling to real-life outcomes. In this structure, Levels 1–3 represent methodological choices and model components (state of research and state of land use), while Level 4 illustrates the objectives these models aim to address. The arrows in the figure represent general linkages between levels rather than fixed causal feedbacks; the bottom feedback arrow does not imply a direct relation between outputs and foundational choices and may be interpreted simply as part of the broader iterative modelling cycle.

Level 1 focuses on the foundational aspects of modelling, this level clarifies the underlying methodological choices, assumptions, and structural characteristics of models [69].

Level 2 compiles specific data types categorised as data types play a key role in the performance of a land use model [37]. The categorisation is split into temporal terms (long-term trends, seasonal variations, climate change scenarios, historical data), geospatial terms (remote sensing, spatial analysis, geospatial modelling), catchment model types (hydrological, watershed, food environment interactions, agriculture, forestry), and physical attributes (biophysical, non-biophysical).

Level 3, the core of the research, has two layers. The inner layer examines social (consumption patterns, cultural differences, community coherence, international understanding), economic (supply chain, production costs, technological advances), and environmental (water quality, soil quality, emission production, greenhouse gas intensity of practices) considerations These factors are influenced by broader dynamics, including agricultural production under land use type, policy and governance, and environmental awareness and compliance [37]. However, land use itself is not only shaped by these external forces but also acts as a driver of environmental impacts, while simultaneously responding to technological advancements like improved machinery. The interactions are multidirectional, with land use emerging as both a consequence of and a contributor to political, cultural, and ecological processes.

Level 4 focuses on the outputs, which are the food environment interactions objectives: improved profitability, reduced emission production, and increased land productivity [4]. These objectives are informed by the insights gained from the processes analysed in Level 3. The framework illustrates a cyclical and interconnected flow, where outputs can feedback into refining future model drivers, inputs, and processes. This fosters a continuously adaptive and responsive approach to land use management, ensuring practices are economically viable and environmentally sustainable, and effectively linking academic research to practical outcomes in the food environment interactions sector. The framework aims to highlight the potential value chains in the modelling process and the most prominent players in the development of catchment models. Improved models enhance policymaking by clarifying economic and environmental trade-offs. Better land-use models have been found to can lower property taxes, increase field rental rates, and support high-value crops like food and biofuels [29]. This bridges the gap between scientific models and governance, helping policymakers align sustainability with economic viability.

A similar process of classification of models is used where they split their conceptual framework into three main layers [73]: Core principals, modelling concepts and modelling outputs. An alternative approach has also been applied where they consider each individual stage of the modelling process and create a conceptual framework for each [55]. Although comprehensive, the approach used is more applicable to the area of land use as is seen in Fig. 1. This allows for a more easily accessible overview for the reader. While land use itself is a straightforward process, the decision-making aspect of the topic and the influences on land use are not as clear. Therefore, by outlining a clear pathway from academic formulation to the practical application of the land use scenarios, this allows for a simplified perspective on the convoluted topic. Land use modelling aims to act as a policy instrument, and this framework aims to help to establish how academia and land use models can influence policy.

## Method and data

This systematic literature review differs from a general literature review in that it adopts a more replicable, scientific, and transparent process by using a detailed methodology designed to minimise bias through in-depth literature searches. This creates an ‘audit trail’ of the academic’s decisions, procedures, and conclusions [68, 70]. In this section the methodology used in the systematic review is described. Recognising the prioritisations outlined above, we have performed an analysis based on a 5 step approach to conducting a systematic review [41], as follows; The first step involves formulating and framing review questions prior to starting the review, clearly defining the issues and key concepts to be addressed. Following the crafting of the research question and objectives as outlined in the introduction of this paper. The next step involved finding the relevant studies to the topic the most representative papers in line with the objectives of the research. The search strings used are as follows: ‘agriculture catchment simulation model’ ‘farm level spatial simulation model’ OR ‘agriculture spatial simulation model’ OR ‘Spatial model agriculture economic’ OR ‘Microsimulation model agriculture economic’ OR ‘food production analysis land use agriculture economic model’ OR ‘A land use change model combining social factors with physical landscape factors’. Although a large number of papers papers appeared through the initial search, not all were relevant to the research question Papers were firt screened by title and abstract to determine whether they addressed land-use models and food-environment interactions at localised/catchment scale. Studies that did not fit the criteria as purely conceptual,, not model based or unrelated to agriculture, economics or the environmental dimension were excluded at this stage. Papers that met this inclusion criteria and were inclusive of the topics seen in appendix A, were then reviewed in full text, with additional exclusions applied where methodogy lacked transparency, or the model type was not comparable. The full text review and data extraction, which would consider several variables, ranging from the key terms, citations, and key topics and subtopics with categorisations such as biophysical, economic, environmental, and social considerations, which are described in Fig. 1, the conceptual framework in more detail in Tables 1, 2, 3, 4, 5, 6, 7, 8, 9, 10, 11, 12, 13 and 14. If each category was conceptually engaged with within the body of the paper, and satisfied the definition outlined for each (see results section), it was coded as being present in the paper. This process saw the elimination of a lot of papers, all of which were critically analysed, which eventually filtered the total number of papers down to 48 papers (*n* = 48) that were the most relevant to the research question. Furthermore, to ensure consistency and to gain the most insight to this niche area of study, the papers which were most aligned with the key objectives of this paper, were explored in more detail, where the author of the work would be added to the search string, authors such as Fezzi and Bateman [25] and Hynes and O’Donoghue [36] fell under this category.

The search was conducted on Google Scholar and was coded on a universal excel sheet, based on the presence or absence of the key variables which acted as the key criteria for the inclusion of a paper in the analysis. The next step involves a summary of evidence, this draws on the presence of the research considerations and identification of trends from the presence or absence of the study characteristics, quality, and effects. These variables are then grouped into a number of umbrella subgroups for meta-analysis. Finally, the findings are interpreted, addressing the issues highlighted in the previous steps. Heterogeneity is examined to determine if the overall summary can be trusted. If not, the effects observed in high-quality studies for making recommendations are considered. Final recommendations are then made based on the strengths and weaknesses of the evidence. In each case, there is an emphasis placed on the macroeconomic and microeconomic split of each consideration, to allow for a better understanding of the localised considerations of each topic.

## Results

To understand how the food environment interaction models interact with the real-life implications influencing agricultural productivity, economic benefit and reduced emission production. This applies the conceptual framework in Fig. 1.

### Level 1: foundational aspects of modelling

This section considers the high-level findings from the scoping research – it should identify the broad trends of the research topic, highlighting the main elements catchment level food environment interaction models are based around.

**Table 1:** Prevalence of different scales of analysis

Scale of analysis	Micro	Meso	Macro	Macro to micro (top down)	Theoretical
Total	21	4	20	1	9

The purpose of these findings is to explore the different scales of analysis employed in food environment interactions research, ranging from micro-level to theoretical approaches. The findings assume that micro-level analysis, which focuses on localised, small-scale dynamics, is essential for capturing the detailed, granular interactions within food environments. Meso-level analysis, bridges the micro and macro perspectives, analysing processes at a regional or intermediate scale, which is crucial for understanding dynamics that are too broad for micro analysis but not fully global. Using a resolution finer than the “true” resolution in environmental analysis can lead to different outcomes [6]. Macro-level analysis plays a key role in understanding overarching patterns and policies that affect food systems at a broad scale. The “macro to micro (top down)” approach, is notable for its ability to translate global trends into local impacts, offering insights into how large-scale forces influence small-scale realities. Theoretical analysis, meanwhile, provides a conceptual framework for understanding the interplay of various scales without necessarily being tied to empirical data.

Many models require linking different types of datasets through statistical methods. Spatial microsimulation models typically rely on small-area census data, which is available in most countries. Examples of models in the UK utilize the UK Census Small Area Statistics (SAS) [5, 2]. Similarly, the Australian Spatial MSM model relies on the Australian population census, while the Irish SMILE household model employs small-area population statistics from the Irish census [54]. Table 1 shows that micro and macro analyses are the most prevalent, indicating that research in this field tends to either focus very specifically on local contexts or broadly on global systems. The predominance of these two scales reflects the need to either deeply understand individual elements of food environment interactions or to look at them from a high-level perspective that considers economic, social, and environmental policies on a broader scale. The findings suggest that while both micro and macro perspectives dominate, the other scales—meso, top-down, and theoretical—are equally important for bridging the gaps between local, regional, and global understandings. The presence of theoretical work indicates a continuing need for frameworks that can conceptualize the intricate interactions within food environment interactions beyond empirical boundaries.

The literature reviewed underscores the dominance of detailed micro- and expansive macro-level analyses, highlighting a focus on capturing either specific localised dynamics or broad-scale systemic trends. This multi-scalar approach is vital for addressing the complexities of food systems research, considering both detailed, context-specific factors and overarching, systemic influences. However, the variability in approaches requires more in-depth exploration into the broader methodologies that link these different levels of analysis. Catchment scale models are more applicable for certain topics of integrated land use modelling and offers a methods such as decision trees can be employed for selecting the most appropriate integrated modelling approach under standard application [40].

**Table 2:** Classification of the research application of models

Application of models	Income Distribution	Rural Development	Climate	Economic policy	Carbon / Resource distribution
Micro	8	8	4	9	4
Macro	10	9	9	16	8
Total	18	17	13	25	12

This analysis focuses on the practical application of each paper within the context of land use, highlighting key drivers like income distribution, rural development, climate, economic factors, and resource distribution, including carbon.

The results assume that land use modelling is significantly influenced by a combination of economic factors, climate considerations, and resource distribution. Integrating multidisciplinary perspectives, enhances the accuracy and relevance of these models [43]. Key terms are defined as follows: Income Distribution is the spread of income across a population, influencing land use through varying economic power. Rural Development refers to improving the quality of life in rural areas, linked to land use policies. Climate relates to long-term weather patterns impacting land use decisions. Economic Factors include monetary considerations and policy implications that shape land use outcomes. Carbon/Resource Distribution involves the management of carbon emissions and natural resources, crucial for sustainable land use.

The analysis reveals that economic considerations are predominant in food environment interactions related land use modelling, with 52% of reviewed papers (income distribution, rural development and economic policy) emphasizing these drivers. This trend highlights the importance of economic and resource factors in shaping policies and practices related to land use and rural development. This stresses the need for a multidisciplinary approach in understanding land use change [30]. Across the application of papers, the trends reveal more presence of macro level orientated papers, especially as they relate to the economic policy and income distribution applications. This trend and the associated implications are explored in more detail throughout economic scenarios and discussion section of this chapter.

**Fig. 2:**
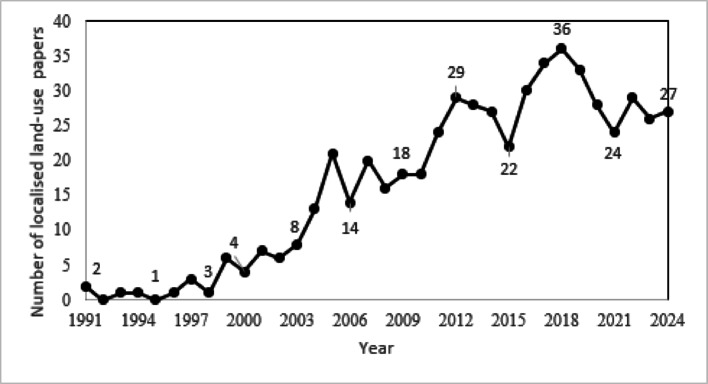
Number of localised land use papers published by year (Scopus)

The trend analysis of publications in the field of land use modelling at the catchment scale with a focus on agriculture, based on the search string TITLE-ABS-KEY (land AND use AND model) AND TITLE-ABS-KEY (catchment AND scale) AND TITLE-ABS-KEY (agriculture) AND PUBYEAR > 1979, reveals several notable patterns. This analysis was conducted using Scopus’ search term analyser, which allows researchers to examine the frequency and distribution of publications over time, offering insights into the evolving interest and development within specific research domains. For this trend analysis, Scopus was chosen because it provides a cleaner handling of search strongs and tools for visualising publication frequency over time. In contrast, Google scholar was used for the systematic review dataset (48 papers) because it casts a wider net, capturing non-indexed and other literature that Scopus excludes. It is important to note that the Scopus trend analysis is explanatory and contextual, and the numbers shown here should not be compared directly with the 48 papers included in the systematic review. As such, .the interpretation of Fig. 2 remains illustrative and is presented with caution, acknowledging the limitations of each database.

Figure 2 above displays the papers published in Scopus over the last 4 decades. This graph reveals growth in the area that has been steadily increasing since 1990’s. Although a steady increase since 1991, placing a focus on the last decade (2014–2024), the graph shows an initial upward trend in the number of papers published, peaking in 2018 before featuring a fluctuation in subsequent years. This upward trend is indicative of a growing interest and suggests an increase in funding and/or research activities in this field. The number of publications increased significantly from 22 in 2014 to a peak of 36 in 2018. This peak represents the highest number of papers published in any year during the entire period. This period reflects an increase within academia’s efforts to address complex, large-scale land use challenges that intersect with agricultural practices.

After 2018, the trend shows a noticeable and consistent decline, with publications dropping to 24 by 2020. This decline may reflect a shift in research priorities, the culmination of major research projects, or the impact of global events such as the COVID-19 pandemic, which diminished research activities and output across many fields [63]. Despite this decline, the field has shown resilience with a modest recovery, recording 27 publications in 2024. The publication trends from 2014 to 2024 reveal both the dynamic nature of research in land use modelling at the catchment scale and the potential influences driving these trends.

The trend indicates a healthy but fluctuating field of study, with periods of intense research activity likely responding to external funding, global environmental concerns, and technological advancements. The recent uptick in publications by 2024 hints at a potential resurgence or stabilisation of interest in this area, possibly driven by ongoing global challenges related to land use, climate change, and sustainable agriculture.

Future research could benefit from closely monitoring these trends and understanding the underlying causes of the fluctuations to better anticipate changes in research focus and funding opportunities. Additionally, analysing the content of the publications from this period could provide deeper insights into the evolving methodologies, key findings, and emerging topics within the field. It is noted that academic research does play a part in shaping policy [33]. Therefore, an increase in participation and addition of published papers in this field is a positive sign.

### Geographic scope—availability of granular data and its impact on localised land use modelling

**Table 3:** Geographical scope at micro level

Geographic scope	Frequency
UK	8
Australia	5
Ethiopia	1
Kenya	1
Ireland	4
Netherlands	2
Italy	2
China	1
France	2
United States	2
Chile	1
Belgium	1
Spain	1
Ukraine	1
Ecuador	1

**Table 4:** Geographical scope at macro level

Geographic scope	Frequency
Continent (Europe)	7
Whole world	8

Tables 3 and 4 above displays the geographical scope of the papers gathered throughout this research process. A broad Geographical spread allows for widespread considerations throughout the process. The trends reveal that the UK and Australia are the leaders in the space. However, it’s notable that only 33 papers explicitly identify a country or region, while 15 lack such specificity, potentially complicating analysis. This is because several of the analyses were conducted at a macro level, with a across larger regions and a number of countries. Agriculture and food environment interaction models are a very geographic specific topic, so it is important that this research presents a widespread presence as much as the literature can allow it.

As estimation techniques typically involve combining households in surveys with small-area census data, this data may not always be available in different countries. This acts as a major barrier to the area of localised land use modelling [5]. The results in Table 3 suggests that the lack of such granular data can be influenced by the geography of an area and could be as a result of a number of factors such as lack of funding, lack of governance structures and possible that this lack of granular data. This will be discussed in more detail in the results and discussion section of the paper.

Spatial microsimulation models often integrate census and survey data to generate synthetic populations within localised geographic areas [36]. While these models perform well when benchmarked against real-world data, they may struggle to capture the full spectrum of spatial variations evident in observed data, highlighting a challenge in achieving comprehensive accuracy across diverse contexts. Another finding was the high occurrence of geographic papers at a macro scale that engage with a broad geographical area. A common approach to food environment interaction models when considering policy implications is to run the analysis at a macro level across EU countries to investigate the impact of a policy [77, 67, 59]. However, in doing so, results in less granular findings.

### Level 2: data inputs

This section highlights the core data types in the research topic. By revealing trends amongst the data, this section aims at identifying the most popular types of land use models, what the key components are and how researchers frame them.

**Table 5:** Temporal trends in modelling of food-environment interactions

Temporal terms	Long-term trends	Seasonal variations	Climate change scenarios	Historical data
Micro	10	1	5	12
Macro	10	3	9	9
Total	20	4	14	21

The purpose of these findings is to analyse the temporal terms used in food environment systems research, aiming to understand how agricultural trends are identified and which approaches are most employed. The analysis assumes that focusing on temporal aspects like long-term trends and historical data is essential for comprehending the sustainability and resilience of catchment systems over time. Long-term trends help assess the ongoing viability of these systems in specific geographical areas, while historical data allows for comparisons between the current state of a catchment and its past, using previous conditions as a baseline for measuring changes. Climate change scenarios appear less frequently in the literature, representing only 24% of the papers, suggesting that this aspect is not as commonly explored. More specifically, there is less micro scale models considering climate change scenarios. Dong et al. [21] suggest this is because of the uncertain nature of climate change, with additional data prediction challenges at the smaller scale levels of analysis. Seasonal variations are the least represented, indicating that short-term fluctuations receive less attention. The review reveals that while long-term and historical data are prioritized, there is a gap in the literature regarding the integration of climate change scenarios and seasonal variations, highlighting potential areas for further research to fully capture the complexity of food environment interaction systems.

**Table 6:** Application of Geospatial methods

Geospatial terms	Remote sensing	Spatial analysis	Geospatial modelling
Micro	6	10	18
Macro	10	13	10
Total	16	23	28

The purpose of the geospatial terms used in food environment interactions research, with a focus on understanding the spatial dimensions of the research questions and identifying the most popular methods employed by leading researchers. It is assumed that engaging with geographical model systems is essential for accurately capturing the spatial relationships and patterns within food environments. This analysis further assumes that breaking down geospatial methods into specific categories—such as remote sensing, spatial analysis, and geospatial modelling—provides valuable insights into how researchers approach these spatial questions. Remote sensing refers to the acquisition of information about an area without direct contact, typically through satellite imagery or aerial photography. Spatial analysis involves examining the spatial relationships and patterns within data, often using statistical methods to uncover trends. Geospatial modelling integrates these approaches to simulate and predict spatial phenomena within food environment interactions.

The comparison across these three methods has revealed that geospatial modelling is the most popular, specifically for micro level papers accounting for 65% of all geospatial papers in this analysis. This contrasts with the remote sensing and spatial analysis, which present more in favour of the macro scale analysis. This suggests that researchers prioritise comprehensive modelling techniques to capture and predict spatial dynamics within food environments. These complexities are often highlighted in the research which requires comprehensive modelling techniques to best address the multi-faceted nature of the subject [10]. Remote sensing, while valuable, appears less frequently, indicating that direct observation methods might be used more selectively or in combination with other approaches. The findings highlight the importance of geospatial modelling and spatial analysis in addressing complex research questions and suggest that integrating these methods can provide a more robust understanding of food environment interaction systems.

**Table 7:** Diversity of modelling approaches in food environment interaction research

Catchment Model types	Hydrological	Watershed	Food environment	Agriculture	Forestry	Other land use
Micro	7	3	3	14	1	0
Macro	3	1	3	14	1	2
Total	10	4	6	28	2	2

The purpose of these findings is to analyse the types of catchment models used in food environment research, with a focus on identifying the diversity of models and the gaps in the literature. Key terms are defined as follows: hydrological models simulate the water cycle within a catchment, watershed models focus on the area of land that drains into a common outlet, food environment models analyse the interaction between food systems and the environment, agricultural models examine the impacts of farming practices, and forestry models look at the effects of forest management. Other land use models encompass a variety of other environmental applications.

The results reveal that agricultural models are highly represented, making up 53% of the sample papers, which aligns with the research’s focus on food environments. Across the macro and micro scales, all considerations are relatively even between the two, with the exception of the hydrological and watershed models. This is as a result that water components tend to operate at very different spatial and temporal scales, requiring a need for more specific data sets at a more granular scale [78]. However, the unexpected prevalence of hydrological and watershed models suggests that these water-based approaches are increasingly being applied in food environment research, potentially due to overlapping interests in resource management. This diversity of modelling techniques underscores the adaptability of these models to various environmental challenges. Despite this, the analysis also highlights a clear lack of literature specifically focused on localised food environment models, even though the search terms were designed to capture as many of these models as possible. This gap suggests that there is significant room for further research in developing and applying food environment models at more localised scales. Utilising more than one approach to a catchment model may assist in addressing this gap. Doelman et al. [20] take 5 different approaches to the shared economic pathways using their own ‘IMAGE 3.0 integrated assessment model framework’. Through the combination of models, this allowed the researchers to account for a wider range of variables, painting a more accurate picture of the food environment interactions.

**Table 8:** Prevalence of biophysical vs. non-biophysical models of food environment interactions

Physical attributes	Biophysical	Non-Biophysical
Micro	15	3
Macro	19	3
Total	34	6

Intertwined with the physical presence is the physical attributes of the models. The purpose of these findings is to examine the role of biophysical versus non-biophysical models in food environment interactions microsimulation research. This distinction is critical for understanding the different angles researchers take when analysing food systems and the implications for economic and environmental connections.

The findings assume that biophysical models, which prioritise natural elements like ecosystem interactions and landscape variables, are essential for understanding how environmental changes impact food systems. It is also assumed that non-biophysical models, which emphasise economic and social factors, are crucial for analysing human behaviour and policy impacts. The assumption is that both perspectives are necessary for a comprehensive understanding of the food environment. Biophysical models focus primarily on natural elements, such as ecosystems, climate, and landscape variables, often placing economic and social considerations secondary to environmental factors. In contrast, non-biophysical models prioritise economic and social factors over environmental variables, focusing on how human actions and policies influence food systems.

Table 8 shows that biophysical papers are more prevalent, indicating a strong emphasis in the literature on understanding natural processes and their implications for the food environment. The majority representation of biophysical models reflects the importance of physical and biological aspects in modelling the effects of environmental changes, such as climate variability and soil health, on food production. The findings suggest that while biophysical factors are seen as primary drivers, non-biophysical models are equally important for providing insights into how economic and social dynamics affect food systems. This division reveals the need for integrating both perspectives to fully address the complexities of food environment interactions.

The literature reviewed highlights the dominance of biophysical models, as these are often considered essential for capturing the natural processes driving food systems. The integration of both biophysical and non-biophysical perspectives is necessary to create a well-rounded understanding of how different factors interact within food environment interactions. However, the spatial variability of biophysical models is noted throughout the literature and requires in depth engagement with the specific area to unveil the themes throughout [72]. The biophysical papers are also weighted in favour of the macro scale models, this allows for an easier data collection process because of common data sets that feature similar soil types, production systems or data sets belonging to the same administrative region. This is explored in more detail in the following sections through levels 2 and 3 of the analysis.

### Level 3: processes and core analytical components

This level examines social, economic and environmental factors, while considering the outer influences of policy and governance, agricultural production method and environmental awareness and compliance. This section identifies the drivers of impact on research and furthermore the drivers of change in each study. The split of variables allows for an understanding of the influences on impacts relating to the real-world issues and the overarching aim of improving profitability, decreasing emissions and improving productivity amongst food environment agricultural areas. By differentiating between the macro and micro models, this offers another layer of analysis, highlighting the granularity of engagement at a farm level.

**Table 9:** Economic drivers of agricultural Decision-Making

Economic Considerations	Supply chain	Production costs (input)	Labour costs	Government subsidies / incentives	Cost effectiveness	Farm profit	Economic scenario running
Micro	9	8	5	8	8	9	8
Macro	8	10	5	11	9	14	10
Total	17	18	10	19	17	23	18

The purpose of these findings is to analyse the economic considerations that influence decisions made by farmers to maximize economic benefits within the agri-environmental sphere. The analysis assumes that economics is a central driver in agricultural practices, affecting everything from production costs to farm profitability. Economic considerations are defined by key terms such as supply chain (the sequence of processes involved in the production and distribution of a commodity), production costs (expenses related to inputs like seeds, fertilizers, and machinery), labour costs (wages and associated expenses for farmworkers), government subsidies/incentives (financial support from the government to encourage certain farming practices), cost-effectiveness (the efficiency of production relative to its cost), farm profit (the financial gain after all expenses are accounted for), and economic scenario running (modelling potential economic outcomes based on varying factors).

The results indicate that government subsidies/incentives, supply chain factors, and production costs are similarly represented, each appearing in 17 and 19 papers respectively, suggesting they are central to agricultural input considerations. Farm profit, appearing consistently across studies, is highlighted as a crucial factor, emphasizing its importance in determining the success of farming operations. Levers et al. [45] discuss the significant role of socio-economic conditions in shaping farm management decisions, noting that while climate conditions are fundamental, farm profit is the downstream effect that ultimately determines a farmer’s success. Their study, conducted at a regional level, suggests that more localised models could enable researchers and policymakers to engage more directly with issues related to farm profit and broader economic considerations [48]. This is highlighted further in the micro and macro split of each topic, with macro level papers outweighing the micro level papers (apart from the supply chain). This points to a potential gap in localised economic modelling within the field, highlighting an area for further research.

**Table 10:** Food environment characteristic considerations

Food Environment	Food access	Food availability	Food security	Crop yield	Food supply chain
Micro	1	5	3	12	12
Macro	0	1	2	14	11

The purpose of these findings is to analyse the presence and treatment of food environment topics in the reviewed literature, focusing on six key areas. It is assumed that food environment considerations, such as food access, availability, and security, are fundamental to understanding agricultural and food systems. The analysis further assumes that models addressing crop yield and food systems should be well-represented due to the key role in crop yield in the output function in the food environment models. Food access refers to the ability of individuals to obtain food, food availability concerns the supply of food within a certain area, food deserts are regions with limited access to affordable and nutritious food, food security involves the consistent availability and accessibility of food, crop yield measures the amount of agricultural output, and food supply chain encompass the entire process of food production, distribution, and consumption.

The results show that while food systems are a crucial component encompassing the whole food production process within the food environment, they are mentioned in only approximately half of the reviewed papers. Ramankutty et al. [62] emphasize the importance of integrating breeding with agro-ecological methods to improve food security and suggest that more comprehensive research could enhance model quality, reducing the need for broad assumptions.

In contrast, Fezzi and Bateman [25] present a robust spatially disaggregated structural econometric model of agricultural land use and production. However, the data used for this model dates back to 1989, highlighting that while well-built models can endure over time, there is a noticeable lack of updated, high-quality models in the field. This gap presents challenges, particularly in adapting to technological advancements and creating user-friendly interfaces. The findings suggest that despite the enduring relevance of certain models, there is a need for newer, more sophisticated models to address current issues in the food environment more effectively. The presence split of micro and macro models are relatively even across the topic, with a stronger presence of micro economic models in food availability models as analysing food availability at a macro level would not allow the same depth of analysis. However, Capone et al. [16] believe that both play a part, stating that at the macro-level some useful insights about food affordability are provided by cereals imports dependency ratio and the values of food imports over total merchandise exports while food affordability score, food consumer price index, while micro level analysis allow a good appraisal of economic accessibility to food at household level.

**Table 11:** Agricultural greenhouse gas emissions in modelling Food-Environment interactions

Emission Production Considerations	Enteric fermentation	Urea application	Liming	Agricultural soils	Manure management	Nutrient emission dynamics
Micro	2	0	0	14	3	0
Macro	1	1	1	17	4	8
Total	3	1	1	31	7	8

The findings highlight the importance of considering emission production in modern food environment catchment models, particularly considering greenhouse gas (GHG) emissions associated with agricultural practices. It is assumed that understanding the sources and impacts of these emissions is crucial for developing effective models and policies aimed at reducing the agricultural sector’s environmental footprint. The analysis assumes that the named emission sources are key factors in this context.

Enteric fermentation refers to methane emissions produced during digestion by ruminant animals. Urea application and liming refer to agricultural practices that release nitrous oxide and carbon dioxide, respectively. Agricultural soils contribute to GHG emissions through the release of nitrogen oxides, while manure management concerns the treatment and storage of animal waste. Nutrient emission dynamics encompass the broader interactions and movements of nutrients, particularly nitrogen and phosphorus, within agricultural systems [23, 24]. Fuel combustion involves the burning of fossil fuels for agricultural machinery and operations but was omitted from results due to an absence of presence in the literature.

The results reveal a significant gap between academic research and current data on emission production. While enteric fermentation is the largest source of agricultural GHG emissions according to the Environmental Protection Agency‘s (EPA) 2022 data [71]. Moreover, it is underrepresented in the research, appearing in only 6% of the reviewed papers. In contrast, agricultural soils are a dominant focus in the literature, appearing in 65% of papers, despite the EPA attributing only 18.8% of agricultural emissions to this source. This discrepancy suggests a lag in academic focus and raises concerns about the alignment of research with the most pressing environmental issues.

It is essential to have an accurate representation of the real environmental influences on emissions output to understand the impact of different farming practices on the environment. This needs to be recognised in the literature and built into the models. Hynes et al. [26] provide a relevant example by combining economic and biochemical models to assess GHG fluxes, carbon stock changes, and nitrogen budgets in European agricultural soils. Their findings support the enhancement of nitrogen and carbon fluxes to boost soil productivity. However, challenges related to data accuracy, the need for downscaling, and the paper’s age (over a decade old) highlight ongoing issues in this research area. The lack of recent studies in this field underscores the necessity for updated and precise research to inform policy and guide global efforts toward reducing agricultural emissions. Looking at specific emission production avenues, the macro-level analyses are outweighed by the micro level. This gap is particularly apparent in the nutrient emission analyses.

**Table 12:** Social influences on agricultural Decision-Making and land use modelling

Social Factors	Farmer age	Cultural/Social influences/Community Dynamics	Education levels
Micro	3	7	4
Macro	2	10	0
Total	5	17	4

Social factors significantly influence decision-making in agriculture, with this analysis focusing on three key elements: farmer age, community dynamics, and education levels. These factors collectively appear in a total of only 54% of all the reviewed literature, indicating a notable gap in research considering the central role of the farmer in agricultural productivity. “Farmer age” refers to the average age of those managing farms, which can impact innovation adoption and long-term planning. “Cultural/social influences/community dynamics” encompass the social structures and cultural practices that influence farming practices, while “education levels” pertain to the formal and informal knowledge base that farmers draw upon. The inclusion of broad ranging social factors is common in this research area [32]. In this analysis, it was intended to highlight the presence of social factors in similar analysis, rather than focusing on specifics.

The results reveal a concerning underrepresentation of social factors in existing models, despite their recognized importance. This underrepresentation is compounded by challenges in data collection and a lack of standardised units of analysis between social and biophysical aspects, as noted by Wehner et al. [79]. Their study emphasizes that while social factors are critical, they are often considered secondary to physical factors in data collection, leading to less accurate models. The localised focus of the analysis suggests that more granular, micro-level studies could better incorporate social dynamics, but the current lack of data poses a significant barrier. The findings underscore the need for more comprehensive data collection that integrates social factors into land use modelling. This is supported by Skaalsveen [66] who state that interpersonal networks play a crucial role in farmer learning and decision making.

**Table 13:** Policy and governance considerations

Policy and governance	Land-use planning	Environmental policy	Agricultural policy	Governance structures
Micro	9	6	8	9
Macro	14	9	11	10
Total	23	15	19	19

Policy and governance play a pivotal role in shaping agricultural practices, with land use planning, environmental policy, agricultural policy, and governance structures being key areas of focus. This analysis shows that these elements are highlighted in only 48% of the reviewed papers. It is assumed that effective land use modelling must incorporate these policies to accurately reflect real-world scenarios. Land-use planning refers to the strategies and decisions regarding the allocation and use of land, while environmental policy and agricultural policy encompass the regulations and guidelines that govern environmental protection and agricultural practices, respectively. Governance structures relate to the systems and processes through which decisions about land use are made and implemented, involving various stakeholders from farmers to policymakers.

The results indicate that land use planning and governance considerations are the most frequently discussed. Macleod et al. [49] emphasize the need for a unified decision-making approach that integrates national and local policies, highlighting existing gaps in policy integration that lead to conflicting outcomes. Van Delden et al. [72] discuss the challenges of scaling national land use models down to catchment levels, noting the complexity of integrating diverse processes and policies across scales. The overall trend of low representation across these variables suggests that the field is marked by inconsistencies and gaps, which complicates the development of robust, integrated models. The low representation is emphasised further at a micro level where there is a significantly smaller proportion of the papers present in comparison to the macro level papers. The findings highlight the need for more cohesive policy integration and better representation of governance structures in land use modelling, especially at a more localised scale. This is reflected in the data gathered for this paper. Papers that engage with specific policies such as the EU wide common agriculture policy (CAP) engage with regions at a macro level with a lack of engagement with the policy effects at a localised level [28, 57], leading to a lack of granularity in the understanding of the impact of such policies on food-environment outputs.

**Table 14:** Presence and effectiveness of Farm-Specific recommendations in food environment research

Farm recommendation	Reducing fertiliser	Reducing livestock	Convert to alternative farming methods (crop/arable)	Maintain current standards
Micro	6	3	2	3
Macro	2	3	5	4
Total	8	6	7	7

The analysis of farm recommendations in food environment models reveals a lack of focus on advising farmers to optimise economic output or reduce emissions via certain farming practices. Among the papers reviewed where these recommendations were present, only 58% provided actionable conclusions for farmers, highlighting a significant gap in guidance for farmers within the research. Key terms include reducing fertilizer, which involves decreasing the use of chemical fertilizers to lower environmental impact. Reducing livestock, aimed at cutting down on greenhouse gas emissions associated with animal agriculture, converting to alternative farming methods (crop/arable), which suggests shifting to crop production or other sustainable practices, and maintaining current standards, which implies continuing with existing practices that are deemed effective.

The results indicate a clear underrepresentation of farm-specific recommendations in the literature, reflecting a broader challenge within agriculture where the heterogeneity of studies complicates the dissemination of applicable information to farmers (Serebrennikov et al., 2020). The scarcity of recommendations suggests that farmers might struggle to adopt best practices without clear, tailored guidance. Lapple and Van Rensburg [42] identify the state of information gathering as a critical factor influencing whether farmers are early, medium, or late adopters of new farming methods. Their research indicates that late adopters tend to realise less profit, underscoring the importance of timely adoption of innovations in tackling climate change and optimizing farm management.

In the context of food environment agriculture, the timing of adopting new technologies and practices is pivotal for addressing climate challenges. The analysis suggests that developing more localised, micro-level models could offer specific, actionable recommendations tailored to the unique conditions of individual farms, thereby reducing the risk of misleading or inaccurate advice. This approach would enhance the relevance and applicability of recommendations, supporting farmers in making informed decisions that align with both economic and environmental goals. Coronese et al. [19] offer a new approach to micro level land use modelling in their AgriLOVE model that uses an agent-based model designed to capture micro level production patterns, focusing on real-world behaviours such as productivity growth, price shifts, and market concentration. Additionally, the model looks at climate impacts and soil degradation over time, providing a detailed insight into the resilience and vulnerabilities in agricultural systems under varied environmental pressures.

## Discussion

This article summarises the results of the systematic literature review on food environment interaction models in the agricultural economics area. The aim of this paper was to consolidate the findings from existing literature and identify any gaps, especially concerning the consideration of emission production and economic outputs associated with land use models in the agricultural economic area. This section will dive into the deeper nuances associated with the findings from the results section. Moreover, this section will acknowledge any related biases and propose potential directions for future research.

### Data accessibility in land use modelling research

As reflected in the results section in Sect. 4 of this paper, there is a lack of literature specific to microsimulation models in the area. Salmon-Monviola et al. [65] addressed this issue more than a decade before this study, stating that ‘the description of spatial dynamics of cropping systems at fine resolutions is particularly needed’. This is further supported by Lazrak et al. [44] where they say that in agricultural landscapes, methods for identifying and describing meaningful patterns are crucial for understanding the interaction between landscape organisation and ecological processes.

Data accessibility poses a significant challenge in developing food environment interaction models due to the extensive and detailed datasets required, which are often not readily available. Burdett et al. [14] addressed this issue innovatively in their study using the Farm Location and Agricultural Production Simulator (FLAPS). This spatial microsimulation model simulated the distribution and populations of individual livestock farms across the contiguous United States, focusing on domestic pigs. To tackle the data gaps, Burdett et al. developed algorithms to match the farm level data to the U.S. Census of Agriculture. This allowed them to impute unpublished state- or county-level livestock population totals, which were otherwise redacted to ensure confidentiality. Moreover, they used a weighted sampling design to gather data on the presence and absence of farms, ensuring a representative sample despite incomplete data. With the collected data, they developed a national-scale distribution model that predicted the distribution of individual farms at a 100-meter resolution.

This approach by Burdett et al. effectively addressed data accessibility issues, demonstrating how advanced statistical and computational techniques can overcome data limitations and enhance agricultural modelling.

### Trend of once-off papers

A common trend in the literature is the lack of follow-up papers, where models are often used only once and then discarded. This was supported through the search methodology for this paper, where special attention was paid to key authors and leaders in the academic space, to follow up models or associated papers within this search string. This pattern represents an inefficient use of resources, suggesting a need for structures that promote ongoing research using existing models. This approach would benefit both the original and subsequent studies by building on prior work. Hertel et al. [34] highlight a similar issue in their review of global-local-global linkages in economic land-use/cover change models, noting the challenges of making these models accessible for modification and extension by other researchers.

Britz et al. [13] provide a notable example of how to address this issue. Their study integrates two economic models, CLUE and CAPRI-Spat, to explore interactions between economic and geographic dimensions. This approach demonstrates the value of combining and extending existing models, which can be particularly beneficial in complex areas like land-use and food environment catchment modelling. By utilizing previous models, researchers can cast a wider net and save time and resources that would otherwise be spent constructing new models from scratch. Promoting the reuse and extension of models in research not only enhances efficiency but also fosters a more collaborative and academic environment. By implementing structures that facilitate model sharing and improvement can significantly advance knowledge in fields such as economic land-use modelling, ultimately leading to more robust and comprehensive findings.

### Improving georeferencing of micro-level data

A recurring theme throughout the research process was a lack of micro level data in the literature. There exists a plethora of land use models, however, limited number of papers in the literature of food environment microsimulation land use models. Macleod et al., [49] highlights the importance of this localised data level in the topic of sustainable food-environment catchment level models, stating that having data at a catchment level requires a more all-encompassing data set which in turn provides robust analysis of the state of the environment across all biophysical, social, political and economic settings. O’ Donoghue [54] states that microsimulation modelling plays a key role in environmental regulation. As such, improving georeferencing of micro level data is key to informing policy and ensuring the most productive environmental regulation. This is further supported by Carletto [17] who discuss the era of transformation that is ongoing in the area of climate change as it relates to agriculture and the important role that the development of agricultural data systems plays in this. More specifically, the paper discusses the logistical and cost challenges associated with gathering local farm level data for research.

A potential reason for the lack of micro or catchment specific papers may result from the challenges in producing very accurate land use models at a localised scale. Dong et al. [21] conducted a study around land use mapping errors and found that error in the land use studies the influence of errors was ‘highly localised, with concentrations in mixed cropping areas’. The nature of academic writing leans against the notion of potential error in data analysis as a prerequisite to engaging in any study. As such, this may play a part in why localised land use modelling is not more of a common practice in the area of agricultural economics.

### Challenges with unregulated definitions

A significant challenge in microsimulation modelling is the variation in variables used across different countries. This discrepancy necessitates a standardised approach to ensure accurate and comparable simulations. O’Donoghue [54] discusses this in his *‘handbook of microsimulation modelling’*, where he states microsimulation models across different nations “require different variables and in addition the names given to equivalent variables will not generally be the same’ He also stated that ‘a first step in any simulation of common reforms across countries, and any policy swap exercise, would involve somehow mapping and reconciling these differences and equivalencies, and perhaps the renaming of variables into a common scheme.” This point highlights the importance of creating a unified variable framework, which would facilitate more effective cross-national policy analysis. By establishing such a standardized system, researchers can ensure consistency and comparability, thereby enhancing the reliability of their findings and fostering greater collaboration in the field. This was seen in this study, with the different definitions and wordings of ‘catchment’ or ‘localised’ scale models, creating a challenge for collating results and identifying trends in the area.

### Maintainability of economic models

As discussed in the results section, a common theme is once off economic models that are built and never used again. It is in the interest of academics in microsimulation modelling to find a common approach to allow for models to be easier to upkeep, maintain and update without a significant monetary or time investment. Innovative approaches to land use modelling have aimed to balance complexity with practicality, ensuring models are both comprehensive and user-friendly. Gregor [31] built a model called DevelopeR (LUSDR) which incorporates land use behaviour and policy sensitivity desired in a land use model, but has a simple, linear structure and manageable data requirements by operating at individual households and employment establishments level, micro simulating the more complex elements such as the location decisions of land developments. This model demonstrates how intricate land use dynamics can be effectively captured without overcomplicated data demands. By focusing on the decision-making processes at the micro-level, DevelopeR (LUSDR) provides detailed insights into how policies impact land development patterns. This balance of simplicity and detail makes the model accessible for various applications while still offering robust analytical capabilities, thereby supporting more informed and nuanced policy decisions.

### Opportunities and risks in long-term projections

Agricultural production across the globe is under threat due to climate change. Various climate-driven extremes, such as droughts, heat waves, erratic and intense rainfall patterns, storms, floods, and emerging insect pests, have adversely affected farmers’ livelihoods. Future climate predictions indicate a significant increase in temperature and more intense erratic rainfall. However, accurately predicting long-term climatic patterns and extremes remains challenging due to the inherent variability and complexity of the climate system.

Lomax et al. [46] discuss the issues associated with papers in the land use area around projecting land use change into the future, suggesting that the answer to avoiding the resource intensive job is to include future projections with land use and other demographic projections at the beginning of government level planning. In the paper, they present set of open-source models which can be used to undertake the projection work. If this were to be implemented, it would reduce the workload and therefore some of the barriers in place around engaging in microsimulation modelling, encouraging more engagement.

### Integration of social, economic, and biophysical aspects

A challenge in environmental economics is integrating all three aspects of social, economic and biophysical aspects into one model in a concise and accurate matter. Often, to include a in depth analysis of any area which is novel in the field and warrants publishing. Leip et al. [13] recognise the importance of including all these topics in the research. They include a 4-step approach to adequately include all the aspects. These are as follows: First, define calculation units that are homogeneous from an economic and environmental point of view, including the social and biophysical aspects within these considerations. This is to ensure consistency and accuracy across the units of analysis. Following this, breaking down regional agricultural statistics and farm management data from a macro level (such as regional data sets) into these smaller calculation units at farm level for more detailed analysis. Next, they suggest creating a variety of environmental model scenarios and conducting model runs to explore different outcomes and impacts. Finally, combine and aggregate the results from these models to provide a comprehensive interpretation and understanding of the data and findings. This approach allows for a thorough and nuanced analysis of the economic and environmental aspects of the study. This is supported by Gregor [31] who states that ‘the complexity of most land use models gets in the way of their widespread use by planning agencies’. As such, this reduces their impact on policy and influence on land use, which is counter intuitive to the purpose of most agricultural land use papers. Melgar-Melgar and Hall [52] argue that biophysical aspects of economics must be at the centre of agricultural economics, stating that valuing nature and its functions plays a key role in understanding the roots of economic activities, especially in agricultural economics. Equally, as revealed in Sect. 4 of this paper, in biophysical specific papers, the concentration on the biophysical aspects often results in a lack of social considerations. Papers that are all encompassing in their consideration of biophysical, economic and social aspects are best placed to positively influence human behaviour and policy in the realm of agricultural land use microsimulation modelling.

### Essential research infrastructure/resourc

A study from Xie, Zhang et al . [79] highlights that the influence of developed countries in the field of sustainable land use is significantly stronger than that of developing countries. It may be argued that this highlights the infrastructural inequality that exists among land use modelling research. At present, the area of research is a very time and labour-intensive area due to the forementioned complexities associated with the data gathering and transcribing into models. Environmental considerations as it relates to agricultural and food production globally is a universal topic with the common objective of reducing emission production and promoting most efficient farming methods. As such, inequalities in gathering information and collating research are detrimental to advancing the area of research.

Bishop et al. and Rajabifard et al. [9, 61] all argue that the governance structure of a country can has a significant impact on the state of land use modelling research. This is further supported by Bennett [7, 8], who states that ‘Many nations lack a coherent national approach to land administration’. Further to this, Bennett [7, 8] highlights that nations who lack land administration systems impedes on a nation’s ability to respond to emerging national and global-scale issues such as climate-change. This paper calls for national land administration infrastructures. It may be argued that governing bodies such as the UN can play a key role in influencing this commitment. This, paired with open data initiatives has the potential to change the way in which individual countries and the world engages with land use scenarios and tackles the environmental issues that are faced.

### Engaging stakeholders and policymakers

A paper by Hewitt, Van Delden et al. [35, 40] calls for a simplification of land use models to engage more stakeholders. A case study found that when faced with models, a gap in knowledge between the researchers and stakeholders made it difficult for stakeholders to grasp the information, calling for two layers of output from the models ‘involving both ‘hard’ (quantitative, data-driven) and ‘soft’ (qualitative, humanistic) information flows’. Such techniques can remove a barrier to entry from non-academic stakeholders, resulting in a more accessible research area.

Brunet et al. [12] discuss the phenomenon of ‘actionable knowledge’ that can be challenging in the post research phase, stating that once any findings are unveiled in the area, active engagement by ecologists, land-use planners and nature managers is key to delivering tangible change as a result of land use modelling. To combat these challenges, Brunet’s paper [12] goes on to offer four simple, actionable solutions as follows: measure ecosystem services in specific and relatable units, provide easy to interpret visualisations of results, be attentive to the ‘storytelling’ aspect of the research and gamification to enact a culture of cooperation. Albeit simple suggestions, overlooking this section can be detrimental to the effectiveness and implementation of research findings.

### Role of open data initiatives

Jones et al. [38] review the challenges that face agricultural data systems models, with two of the biggest issues being a scarcity of data for developing, evaluating, and applying agricultural system models and inadequate knowledge systems that effectively communicate model results to society. Food environment interaction models help communicate models to society and offer a real, tangible connection between land and policy. However, the data accessibility issues act as a major barrier to contribution to agricultural knowledge systems.

Open data initiatives present a significant opportunity for agricultural land use modelling at a localised farm level. By making data more accessible, these initiatives can enhance land use modelling, enabling farmers and researchers to develop more precise and effective models. This accessibility promotes innovation and supports efforts to address global environmental challenges. Luna-Reyes and Najafabadi [47] discuss the development of Open Government Data (OGD) programs over the past decade has promised benefits such as improved transparency and accountability. In agriculture, this translates to more informed public policy and better decision-making at the farm level. However, to fully capitalize on these benefits, it is crucial to address technical issues and understand the conditions necessary for successful OGD programs. The simplified process of the US Federal Government in implementing OGD policies highlights the importance of effective governance, leadership, and stakeholder engagement in providing effective and accessible solutions. Future research should focus on these aspects to ensure that these programs are effectively implemented and can support domain-specific needs such as agricultural land use modelling. These initiatives can remove a barrier of entry to agricultural land use modelling and thus become a powerful tool in combating environmental challenges and promoting sustainable agricultural practices with improved transparency and reproducibility.

## Conclusion

This review highlights the complexity and multifaceted nature of land use models, emphasizing their crucial role in bridging academic research and practical applications in agriculture. The study acknowledges the increasing sophistication of these models, driven by advances in computing power and the need for more localised and detailed analyses.

The review identifies significant gaps in the literature, particularly the underrepresentation of localised economic and food environment interaction models. While global models offer valuable insights into broad-scale trends, localised models are essential for addressing specific regional challenges, such as adapting to climate change and optimizing agricultural practices for sustainability and economic viability.

Economic considerations, including production costs, government subsidies, and farm profitability, emerge as central drivers in the reviewed models. These factors are consistently represented across the literature, which emphasises their importance in shaping agricultural decision-making and policy. The study also highlights the role of socio-economic conditions in influencing farm management, suggesting that more localised models could enhance engagement with these economic aspects.

The analysis further reveals that while certain elements like food systems and crop yield are recognized as critical components of the food environment, they are not as prominently featured in the models as expected. This points to a need for more comprehensive research that integrates these factors more thoroughly, potentially improving the models’ ability to predict and manage food-environment interactions effectively. Moreover, the review emphasises the value of using modelling approaches to capture the full range of wide-ranging variables. This combats many of the main challenges in the food environment interactions in catchment models. By combining different types of models, researchers can achieve a more accurate and holistic understanding of these interactions, which is vital for developing effective policies and practices.

In conclusion, the paper calls for a pathway to address the identified gaps in the literature, particularly the need for more localised and integrated models. Future research should focus on refining these models to better account for economic, environmental, and social factors at the catchment/farm level. This approach will enhance the models’ relevance and applicability, ultimately contributing to more sustainable and economically viable agricultural practices. The study highlights the importance of continuous adaptation and refinement of land use models to keep pace with changing environmental and socio-economic conditions, ensuring that they remain valuable tools for policymakers and practitioners in the agricultural sector.

## Data Availability

No datasets were generated or analysed during the current study.

## Appendix 1

### Variables coded

**Table:**

Key terms	Paper Reference
	Importance
	Summary
	Select
	Done
	Citations
	Year
	Application
	Key words
Application	Income Distribution
	Rural Development
	Climate
	Economic
	Carbon
Scale of Analysis	Micro
	Meso
	Macro
	Macro to micro (top down)
	Landscape
Catchment Models types	Hydrological
	Watershed
	Food environment
	Agriculture
	Forestry
	Other land use
Model elements	Time unit
	Time period
	Time length
	Country
	Continent
	Catchment
Model system	Both
	Statistical
	Geographic / Mapping
Geospatial terms	Remote sensing
	Spatial analysis
	Geospatial modelling
Policy and governance	Land-use planning
	Environmental policy
	Agricultural policy
	Governance structures
Scale of Analysis	Micro
	Meso
	Macro to micro (top down)
	Landscape
Temporal terms	Long-term trends
	Seasonal variations
	Climate change scenarios
	Historical data
Physical attributes	Biophysical
	Non-Biophysical
Economic Considerations	Supply chain
	Production costs (input)
	Labour costs
	Government subsidies / incentives
	Cost effectiveness
	Farm profit
	Economic scenario running
Food environment	Food access
	Food availability
	Food deserts
	Food security
	Crop yield
	Food systems
Emission production considerations	Enteric fermentation
	Fuel combustion
	Urea application
	Liming
	Agricultural soils
	Manure management
	Nutrient emission dynamics - Nitrogen/Nitrate/Nitrus Oxide/Phosphurus
Social factors considered	Farmer age
	Cultural/Social influences/Community Dynamics
	Education levels
Farm recommendations	Reducing fertiliser
	Reducing livestock
	Coonvert to alternative farming methods (crop/arable)
	Maintain current standards
